# Analysis of the Ag M_4,5_ EELS edge to study silver nanoparticle corrosion

**DOI:** 10.1111/jmi.13348

**Published:** 2024-08-08

**Authors:** JC Brennan, DA MacLaren

**Affiliations:** ^1^ SUPA School of Physics & Astronomy University of Glasgow Glasgow UK

**Keywords:** corrosion, EELS, ELNES, mechanism, nanoparticle, silver, sulphide

## Abstract

Electron energy loss spectra collected from fresh and corroded silver nanoparticles are compared with those from a number of reference materials, focusing on the M_4,5_ edge. Chemical shifts and changes in the energy loss near edge structure (ELNES) are described and found to be sufficient to distinguish metallic silver from chemically oxidised silver. The measurements, in conjunction with electron energy loss spectrum imaging, are used to assess the mechanisms for atmospheric corrosion of silver nanoparticles. We unambiguously assign the corrosion product under atmospheric conditions to be silver sulphide, but show the reaction process to be distinctly inhomogeneous, producing a variety of types of corroded particles.

**LAY DESCRIPTION**: >Here, we use analytical electron microscopy to track the corrosion of silver nanoparticles and present chemical maps of the corrosion products. We show clear spectroscopic differences between metallic and corroded silver using the M_4,5_ electron energy loss spectral feature, which is not commonly studied. Our study shows that corrosion is due to interactions with sulphur in the atmosphere; and the corrosion is not uniform, but appears to develop from specific points on the surface of the nanoparticles.

## 1 INTRODUCTION

Ag and its compounds find use in areas ranging from medicine to plasmonics.^1^, ^2^, ^3^, ^4^ However, the conductivity, optical properties and anti‐bacterial activity are strongly dependent on the Ag oxidation state,^5^, ^6^, ^7^ and it is well known that Ag corrodes under atmospheric conditions. It is sometimes expected^8^, ^9^, ^10^, ^11^ that the principal corrosion product should be Ag_2_O, similar to the oxidation of most other metals. However, since the earliest thin film studies,^12^, ^13^ oxide formation is not found to be facile under atmospheric conditions; in contrast, the presence of a few parts per billion of H_2_S, under humid conditions, is sufficient to promote the formation of Ag_2_S in both thin films^14^, ^15^, ^16^ and nanoparticles (NPs).^7^, ^17^, ^18^, ^19^ Theoretical studies provide an explanation, through the prediction of an activation energy barrier for dissociative adsorption of O_2_ on Ag, which limits the formation of oxides.^20^ The presence of stronger oxidising agents, such as ozone produced by the action of UV light on gas‐phase O_2_, provides an alternative mechanism for the relatively rare observations of silver oxides.^20^ There is no such barrier predicted for dissociative adsorption of sulphides on Ag surfaces, but several experimental studies indicate that adsorbed H_2_O facilitates sulphide formation, which suggests a mechanism mediated by solvated Ag^+^ and HS^−^ ions, the latter of which are formed when H_2_S dissolves in H_2_O.^13^, ^14^, ^15^, ^19^ A number of studies^13^, ^15^, ^16^, ^19^ indicate corrosion to start with the formation of ‘secondary’ Ag sulphide particles that coalesce in thin film systems to form a corrosion coating. A similar mechanism is proposed in nanoparticulate systems,^18^ and a recent electron microscopy study, using Ag nanoparticles of a few tens of nanometres in diameter, provides direct evidence for humidity‐dependent migration of Ag away from the main particles.^19^ Smaller (<10 nm diameter) secondary Ag particles appeared around the main particles and then corroded to form sulphides. Reduction of humidity levels was shown to reduce the production of secondary particles but may not be sufficient to quench the corrosion of the main particles. It seems that in Ag nanoparticle systems, there is therefore an interplay between direct corrosion of larger particles and the formation, then corrosion, of a secondary particle population.

Here, we present high‐resolution observations of the corrosion of ∼20 nm diameter Ag nanoparticles. Corrosion is found to be inhomogeneous, yielding Ag sulphides upon atmospheric exposure. We use electron energy loss spectroscopy (EELS) to analyse the structure and chemical shift of the Ag M_4,5_ edge, in comparison to spectra collected from a number of reference materials.

## 2 METHOD

Commercially available powders (Alfa Aesar) of AgO, Ag_2_O, AgCl, and Ag_2_S, all of ≥ 99.9% purity, were deposited directly onto holey carbon grids, or (Ag_2_O only) were drop‐cast as a suspension in pure ethanol, before immediate analysis in the microscope as reference materials. 20 nm AgNPs (Sigma‐Aldrich) were also deposited directly onto a copper grid coated with holey carbon, at a concentration 0.02 mg/mL, from a sodium citrate stabilising solution. The ‘aged’ AgNP samples were stored on a similar grid in a low vacuum desiccator, in the dark, for 6 months before analysis and transferred to the microscope under ambient atmospheric conditions – these conditions are sufficient to slow but not quench the corrosion observed. We believe it likely that corrosion requires the slow degradation of the protecting citrate stabiliser, which occurs once particles are dried and no longer held in solution. Thereafter, even trace quantities of sulphides, even in the low vacuum of a desiccator, are expected to react with the nanoparticles.

Spectra collected from the centre of fresh AgNPs were taken as representative of pure metallic Ag; they also compared favourably to the reference pure Ag spectra available within the Gatan *Digital Micrograph* ^TM^ software package. Transmission electron microscopy (TEM) and scanning transmission electron microscopy (STEM)‐EELS measurements for all samples were acquired with a JEOL ARM200cF operating at 200 kV. Spectra were collected with a Gatan 965 Quantum ER spectrometer, typically using a probe convergence angle of 29 mrad, collection angle of ∼35 mrad and a dispersion of 0.025 eV/channel. The energy spread of the cold field emission gun was of order 0.5 eV. The Dual EELS^21^ and log‐ratio techniques were used to collect separate low‐loss and core‐loss spectra and then remove plural scattering. An acceleration voltage of 200 kV was used. The beam current was not measured directly but care was taken to avoid any time‐dependent spectral changes that would be indicative of beam‐induced damage. In most cases, a limiting factor was the rapid build‐up of carbon under the electron beam, producing a strong background C‐K edge that rapidly dominated spectra. These effects can often be minimised by ‘cleaning’ protocols, such as exposure to oxygen plasma or a defocused electron beam, but these were not applied to avoid inducing chemical changes to the nanoparticles. Spectrum Imaging^22^ was used to acquire spectra on a pixel‐by‐pixel basis within regions of interest, typically with (0.5 nm)^2^ pixels. Analysis was undertaken within the Gatan *Digital Micrograph* ^TM^ software.

## 3 RESULTS AND DISCUSSION

### 3.1 EELS edge structure

Although it has a relatively large cross‐section and low energy loss, the M_4,5_ EELS edge of Ag is not commonly studied in either EELS or XANES as it is a broad spectral feature with a gradual onset: transitions from a core d‐state to the unoccupied s‐states immediately above the Fermi energy are spin‐forbidden. These conditions imply a relatively low sensitivity to chemical changes, with a majority of the literature instead considering the sharper L_2,3_ edge excitations, even though they occur at higher energy loss, and so have substantially lower cross‐sections. Nevertheless, we find clear variations in M_4,5_ edge structure that are suitable to discriminate oxidation states and bonding environments.

**FIGURE 1:**
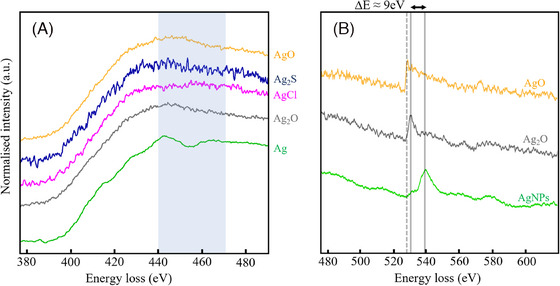
(A) The Ag M_4,5_ EELS edges for (bottom to top) pure Ag, Ag_2_O, AgCl, Ag_2_S, and AgO. For the pure Ag M_4,5_ edge, the most readily identified features are the peak and dip above 440 eV, highlighted by the vertical grey band: these features only appear for elemental silver. (B) O K EELS edges for: (bottom to top) an AgNP sample, an Ag_2_O standard, and an AgO standard. There is a notable shift (9 eV) in the onset of the O K peak for the oxides. All signals have been normalised to their respective maximum edge intensities, to allow for ease of comparison. Some traces exhibit higher noise, due to the short acquisition times used to minimise beam damage. The oxygen signal in the AgNPs spectra is later shown not to be chemically bonded to the Ag and does not affect the Ag oxidation state.

Figure 1 presents experimental measurements of the M_4,5_ EELS edge collected from a sample of pure silver and the four reference compounds, AgO, Ag_2_O, AgCl and Ag_2_S. The references were previously identified as the most likely compounds to form on Ag exposure to atmospheric conditions.^23^, ^24^ AgCl has been observed to form in outdoor conditions, typically in the presence of NaCl.^14^, ^16^ Each Ag M_4,5_ spectrum has been background‐subtracted using a power‐law fit to the preceding data, then deconvolved to remove plural scattering effects. The edge is usually taken as starting at an energy of 367 eV and the pure Ag spectrum agrees well with those of the limited available literature.^25^, ^26^ The levels of noise (here and in later figures) reflect the available signal levels, which were limited by the thickness of the samples and build‐up of carbon under the electron beam, which is typical of particles produced by wet chemical synthesis.

A previous XANES study of elemental silver also shows the characteristic peak and dip above 440 eV,^27^ highlighted by the grey band in Figure 1A. The first peak is assigned to the M_5_ edge, with a 3d_5/2_ initial state and spin‐allowed final state of p_3/2_ character; intensity immediately after the dip is assigned to the M_4_ edge, with a 3d_3/2_ initial state and final state of either p_1/2_ or p_3/2_ character, though the Δ*j* = 0 transitions are predicted to be very weak.^28^ Similar features have also been observed in the M_4,5_ edges of both Rh and Pd, which also have delayed edges involving similar transitions from 3d orbitals to allowed states significantly above the Fermi level.

After chemical oxidation, the sharper features in the Ag M_4,5_ EELS edge are lost and all four of the reference spectra in Figure 1A have a more rounded appearance. There are subtle variations between the Ag^1+^ spectrum of Ag_2_O and the mixed Ag^1+,3+^ states^29^ of AgO but we do not believe these to be sufficient to act as a reliable ‘fingerprint’ to distinguish between these Ag oxidation states in the manner used in most EELS analysis. Similar rounded features have also been presented, without specific discussion, elsewhere; for example, Ref. (26) discusses interfacial oxidation of a silver thin film.

We turn now to the oxygen counter‐ions for the two oxide reference materials, since changes to the O K‐edge are common when a metal oxidation state or bonding changes. The O K edge is observed around 532 eV and so sits on the high energy tail of the main Ag M_4,5_ feature. Spectra collected from the Ag nanoparticles, Ag_2_O, and AgO are presented in Figure 1B and have not been background‐subtracted since the background derives from the Ag edge. The nanoparticle spectra also exhibit a strong oxygen feature because of the presence of significant amounts of oxygen within an encapsulating adsorbate shell of sodium citrate (Na_3_C_6_H_5_O_7_), or its breakdown products under the STEM electron beam. The shell is discussed in more detail below. All the collected spectra tend to also contain very weak features around ∼571 eV that would be consistent with the Ag M_3_ edge.

The oxygen adsorbate shell of the nanoparticle spectrum provides a useful internal reference to assess chemical shifts. Its rising edge is at 532 eV and it has a peak around 540 eV, the details of which will depend on the nature of the molecular adsorbate. In comparison, for both the Ag oxides, the peak associated with the oxygen K‐edge has appeared to shift by approximately 9 eV to lower energy, which is surprisingly large and indicative of a major shift in the electronic structure.^30^ Such a shift is clear evidence of chemical bonding to the Ag and its magnitude makes it easy to analyse, even if the energy resolution of the spectrum is relatively low.

It is known from previous studies of oxides^31^ that as the oxidation state of a transition metal increases, the energy of the O *K* peak decreases, reflecting changes in the nature of the unoccupied density of states at the Fermi energy. This effect can be seen in a comparison between the AgO and Ag_2_O spectra of Figure 1B, where the dashed and solid lines indicate the onset of the AgO and Ag_2_O O *K* edge, respectively. We find a consistent chemical shift of ∼1 eV between the two features, similar to that observed in a different chemical system by XANES and ascribed to excitation into empty molecular Ag‐O π* and σ* orbitals.^32^ In comparison to the large, 9 eV, chemical shift observed for Ag_2_O, the additional shift for AgO is relatively small. However, oxygen will exist in a similar O^2−^ state in both materials and the unoccupied density of states may not differ substantially. In studies of other transition metal oxides, it has been noted that chemical shifts of the O *K* edge depend more on the bonding environment than the oxidation state of the metal ion.^31^

### 3.2 Ag nanoparticles and their corrosion

**FIGURE 2:**
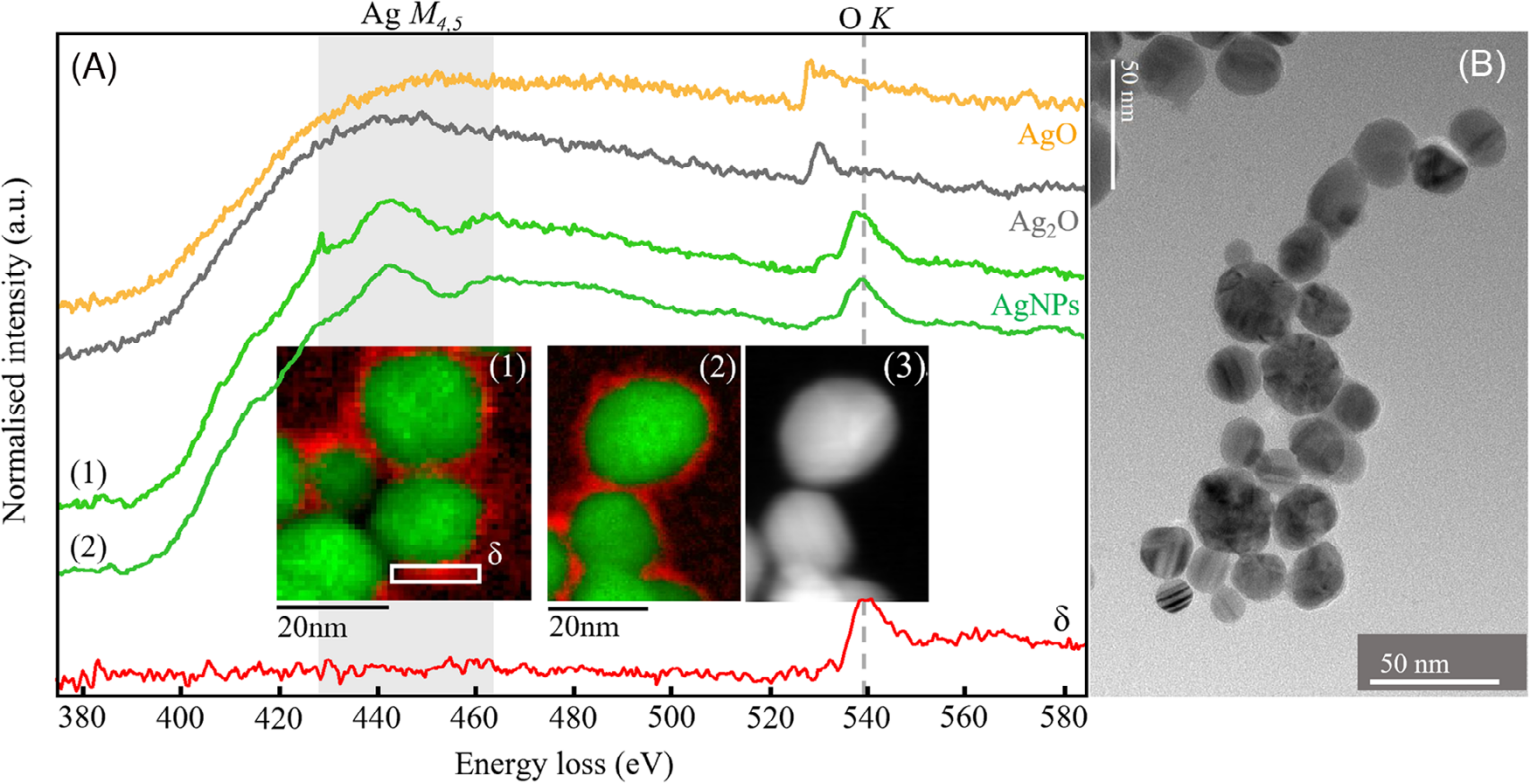
The spectral region surrounding the Ag M_4,5_ and O K edge for four different samples: (top to bottom) an AgO standard sample, an Ag_2_O standard sample, and two AgNP samples. The key observation is the difference in appearance of the Ag M_4,5_ edge between the AgNP samples and the Ag oxides. Even though there are O peaks in the spectra of the AgNPs, and the presence of O in the EELS images, the Ag M_4,5_ edge displays the peak and valley structure of pure Ag, indicating the AgNP sample comprises pure, uncorroded AgNPs, surrounded by a layer of adsorbed O. Furthermore, the position of the O K peak at 540 eV for the AgNP samples, and ∼531 eV for the Ag‐oxide standards indicates that Ag‐oxide compounds are not present in the AgNP samples. The inset images (1) and (2) are EELS maps, corresponding to the green spectral traces (1) and (2), showing the presence of Ag in green and O in red. (3) is a HAADF image of cluster (2). The spectrum labelled δ represents the signal coming from the region highlighted by the white box in (1), containing only the shell around the AgNPs. Here, we see that there is no Ag M_4,5_ edge, and thus no Ag present, while there is a strong O K peak. (B) gives an overview of the freshly deposited AgNPs, showing characteristic size distribution and clustering.

Let us return now to the observation of oxygen in the spectra collected from fresh, citrate‐protected Ag NPs. Overview images and EELS spectra from freshly deposited AgNPs are shown in Figure 2. Figure 2B and C is TEM images of a representative group of freshly deposited AgNPs, highlighting the standard size distribution and clustering typical of our samples. Contrast within these particles derives from diffraction effects, with the particles showing stripes or bands typically being found to be crystalline Ag. The RGB maps in the inset show two AgNP clusters with Ag coloured green and O coloured red. No significant sulphur signals were collected from these particles, while the presence of Ag and O is clearly seen. The O K edge analysed here for AgNP (2) is the same as seen in Figure 1B and looks very similar for AgNPs (1). Importantly, the Ag M_4,5_ edge of the AgNPs has the characteristic peak and valley (highlighted by the grey vertical band), showing it is still pure Ag.

The inset spectrum image maps in Figure 2 are important because they reveal oxygen to reside in a shell (in red) around the AgNPs rather than co‐located with silver: the shell is not a silver oxide. AgO and Ag_2_O spectra are included above the AgNP spectra, for comparison, and neither appear like the AgNP spectra, lacking the characteristic dip around 450 eV. Similarly, the O K peak is shifted to lower energies, as discussed previously. Silver oxide is occasionally considered as the main corrosion product for AgNPs, and the results presented here demonstrate the need for spatially resolved studies of the corrosion mechanism. It is possible that elemental analyses that lack spatial resolution would detect the presence of oxygen within a AgNP sample without distinguishing chemisorbed molecular oxygen‐containing shells from the presence of a silver oxide. Spectra were gathered and summed from within the rectangular region within the first inset to Figure 2A, where only the shell was present. The spectral line from the shell alone shows that there is no Ag present, just a strong O K peak, present at ∼540 eV, unlike the Ag oxide compounds. We therefore conclude that the O present in these samples was not part of an Ag oxide shell. As organic stabilisers are commonly used in AgNP research, their presence and the presence of their remnants and degradation products should be considered in chemical analysis. In the present case, there is a substantial carbon signal from the shell region that is consistent with the presence of citrate or its breakdown products. However, the holey‐carbon support also provides a large carbon background, making the signals difficult to distinguish.

**FIGURE 3:**
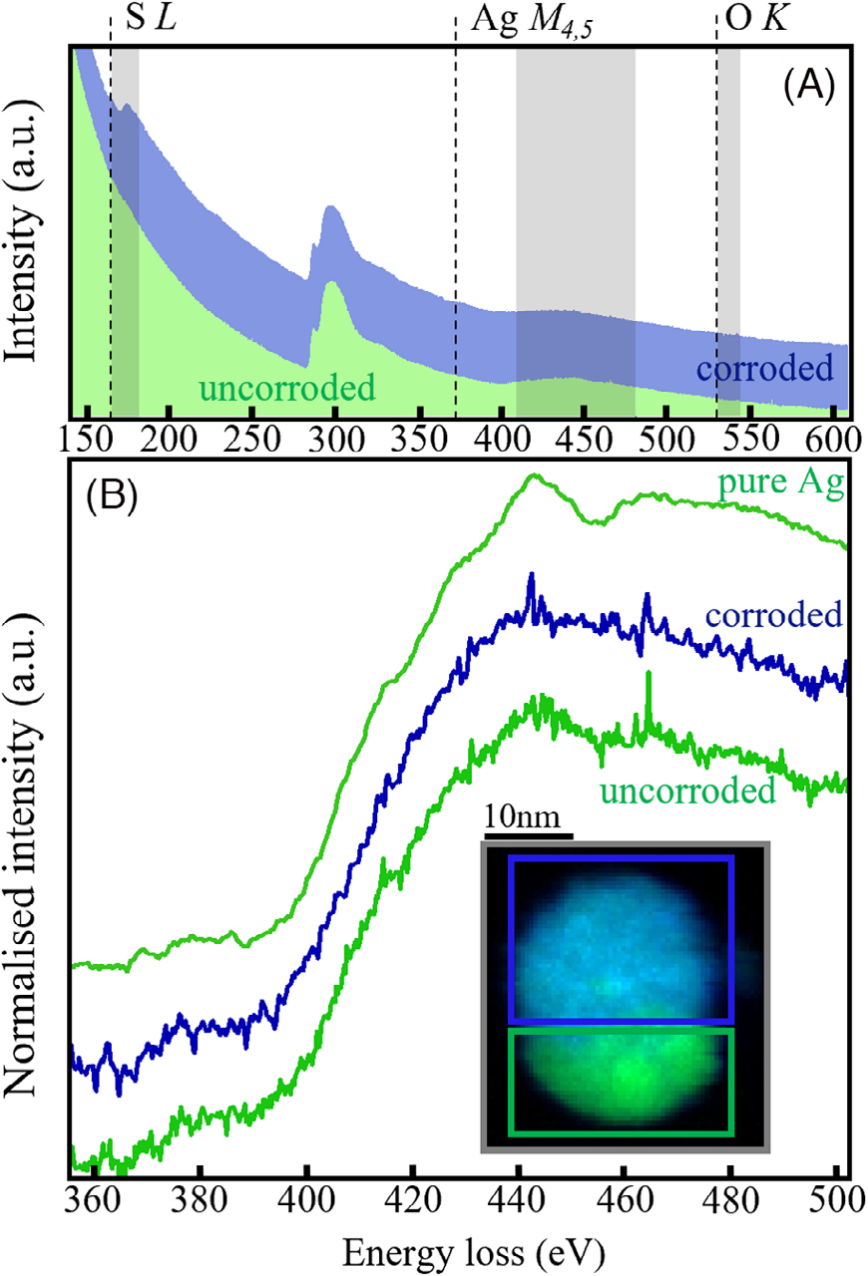
(A) EELS data containing the O K, Ag M_4,5_, Ag M_3_ and Ag M_2_ EELS edges, corresponding to a corroded and uncorroded region of an AgNP exposed to atmosphere. (The large carbon K‐edge, at ∼284 eV, derives from a holey carbon support and is not relevant here.) The AgNP regions examined are shown in the inset of (B). The blue data (top trace) correspond to a corroded region, while green data (lower trace) correspond to the uncorroded portion. (B) The Ag M_4,5_ edge for both regions of the AgNP is highlighted and compared to that of pure Ag. The AgNP is displayed as an RGB spectral image in the inset, with regions containing Ag coloured green, and regions containing S coloured blue. The bottom region of the AgNP is completely green, suggesting pure Ag, an assertion which is confirmed by the bottom spectral line, which contains the characteristic peak and valley at the summit of the AgM_4,5_ edge. The top portion of the AgNP is coloured a mixture of blue and green, suggesting the presence of Ag and S, again confirmed by the blue spectral line which shows the characteristic smooth peak associated with bonded Ag.

In contrast to the fresh AgNPs, an EELS analysis of an aged AgNP is presented in Figure 3. The AgNP was aged for 6 months, as described in the methodology, allowing some level of corrosion to take place. In the inset is an RGB spectral image of the AgNP. As before, regions containing S have been coloured blue, regions of Ag are coloured green, and any O signals would appear red. The top portion of the AgNP is coloured a mix of blue and green, indicating the presence of Ag and S, while the bottom portion is just green, implying a region of pure Ag remains untarnished by S. The Ag M_4,5_ edge from each NP region is shown, with the pure Ag edge added at the top for comparison. The spectra from the NP are considerably noisier, as they were collected with shorter acquisition times; the trends, however, are still evident. The edge from the untarnished lower portion of the NP is shown at the bottom (in green), and the characteristic peak and valley at the summit of the edge is seen, as expected. Above this, the M_4,5_ edge from the sulphur containing region is displayed (in blue), for which the edge has a smooth appearance, indicative of chemically bonded Ag, similar to the Ag_2_S spectrum seen in Figure 1A, and unlike the spectrum for pure Ag.

**FIGURE 4:**
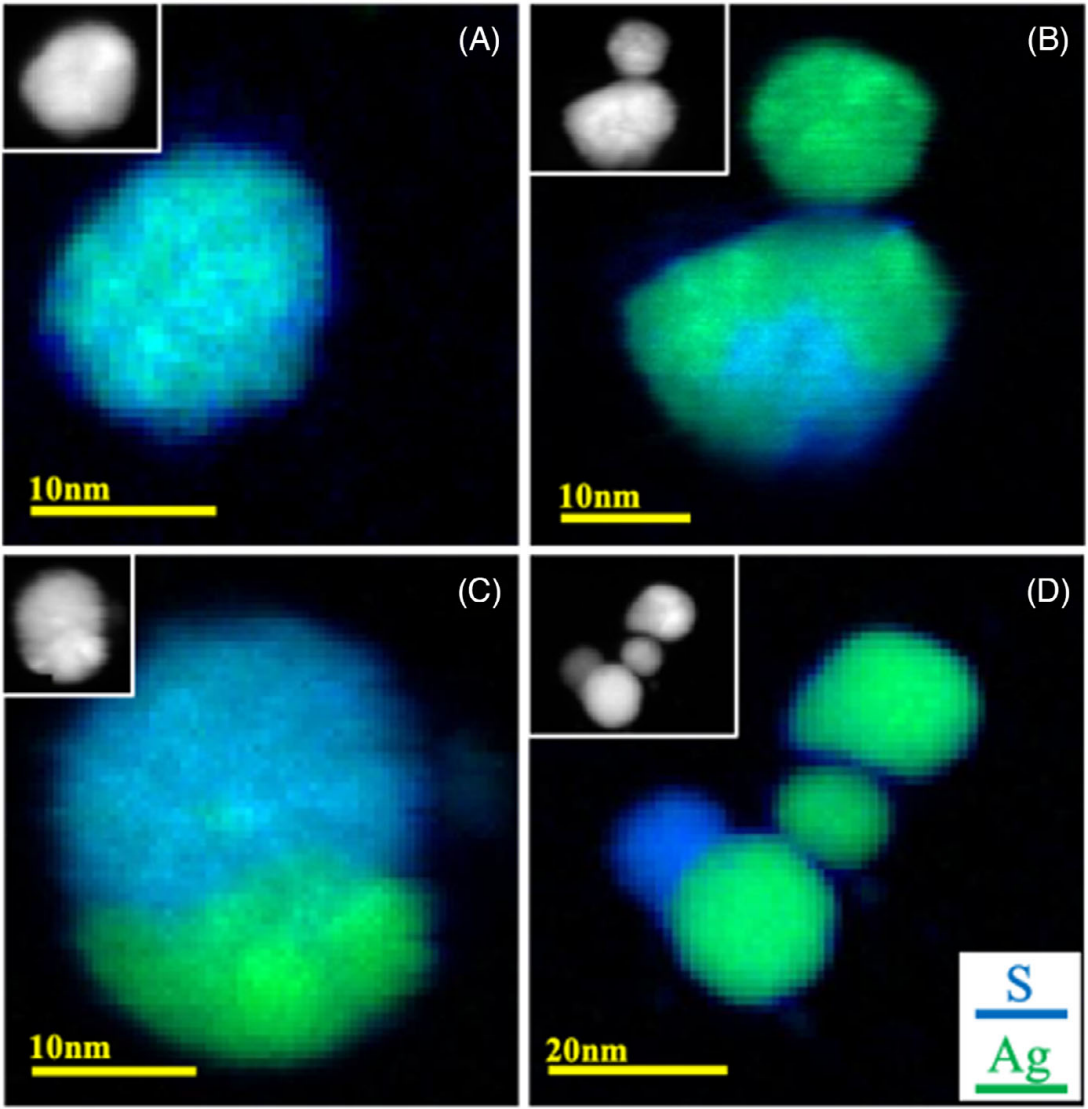
(A)–(D) EELS spectrum images for four AgNP samples, aged for 6 months. Regions in green represents the presence of Ag, while S signals are shown in blue. In the inset for each EELS map are the corresponding HAADF images. The only corrosion product here is Ag sulphide; there is no O present on any of the aged samples. Importantly, the corrosion is inhomogeneous: each nanoparticle has different levels of sulphidation across different regions.

Figure 4 summarises the appearance and chemistry of aged AgNPs, including spectrum images that illustrate the inhomogeneous nature of the reaction to form Ag_2_S. The NPs are generally smaller than those observed previously^19^ to spawn ‘secondary’ particles, which were not observed here. It may be that there is a size dependence for secondary particle formation, whereby an NP needs to exceed a critical diameter before dissolution of Ag^+^ becomes appreciable; alternatively, the humidity levels may have been too low to facilitate this corrosion mechanism. Instead, Figure 4 shows the direct, but incomplete, corrosion of Ag NPs to form Ag_2_S. In each case, the intensity of the inset HAADF images gives an indication of the uniformity of composition, because denser and/or thicker regions appear brighter. Regions of uncorroded, metallic Ag are therefore brightest, and the inhomogeneous composition of particles was evident from HAADF imaging alone.

Figure 4A–D shows different aspects of corrosion, using the same colour scheme as before to indicate the presence of S and Ag (here, there were no detectable O signals). In Figure 4A, a single NP of ∼10 nm diameter has been completely converted to a sulphide. The larger particle in Figure 4B, however, has only partially converted, and the blue region at the bottom of the particle is suggestive of a reaction front that is propagating through the NP from a nucleation point on its surface. The shape and outline of the particle in Figure 4C suggests that it is a compound particle, with an upper spherical NP that has corroded and a lower metallic Ag cap. This is the same particle shown in Figure 3, and its appearance most closely resembles the NPs analysed by Keast,^19^ which tended to appear as clusters of small, reacted particles. This morphology is consistent with a similar mechanism to that proposed for thin film corrosion, whereby secondary corrosion‐product particles appear across an exposed film.^6^ In contrast, the three particles arranged in a line in Figure 4D have hardly corroded at all, with what may be only a thin (∼1 nm) sulphide shell, showing as a slight blue edge to the particles, which appears consistent across all three particles. The spatial resolution of the spectrum images collected here precludes a definitive assignment of chemistry for any thin (∼1 nm) ‘shell’. (The low S EELS cross‐section and rapid build‐up of carbon under the electron beam were also limiting factors.) The formation of an apparently protective shell would be unusual because the proposed mechanism for corrosion of thin films tends to involve migration of S ions into the film and migration of Ag out, which is not self‐terminating.^20^

The inhomogeneous nature of AgNP corrosion likely stems from a number of factors. The first is an inconsistent degradation of the encapsulating citrate shell around each NP, which permits HS_2_ to access the Ag NP and react, perhaps with the subsequent corrosion limited by the diffusion rate of H_2_S through any defects in the adsorbate shell. The ability of molecular adsorbates, or carbonaceous shells, to restrict corrosion is described elsewhere.^33^, ^34^ Secondly, it has long been known that the reactivity of exposed Ag surfaces will also depend on the surface crystallographic termination, with close‐packed surfaces being least reactive.^20^ None of the particles here exhibit faceting or a pronounced geometric appearance that would be consistent with a strong variation in surface energies, which is likely a consequence of adsorbed molecular species. Nevertheless, surface step, kink, vacancy, and other low‐co‐ordination sites can be expected to be more reactive and to offer low‐energy pathways to sulphur ingress as a first reaction step for corrosion. A further consideration is the presence of grain boundaries and dislocations, which offer a combination of strained bonds and low‐coordinated sites are also known to be more reactive. They have been proposed as sites of preferential Ag_2_S nucleation in patterned Ag thin films^18^ and will also help explain the inhomogeneous reactions observed here. More detailed in situ electron microscopy studies of corrosion in real time would now provide useful insight into the detailed mechanisms producing this variety of corroded NPs.

## 4 CONCLUSION

We have analysed the Ag M_4,5_ EELS edge to distinguish bonding and oxidation state changes in silver. The data were used to confirm silver sulphide as the primary product of atmospheric corrosion. We found the corrosion mechanism in nanoparticles to be inhomogeneous, producing seemingly stable nanoparticles comprising a mixture of chemical phases. These observations will have implications for applications employing Ag nanoparticles.

## AUTHOR CONTRIBUTIONS

Both authors were involved in all aspects of the work from data collection and analysis to paper writing.

## CONFLICT OF INTEREST STATEMENT

The authors declare no conflicts of interest.
